# The effectiveness of incentives for research participation: A systematic review and meta-analysis of randomized controlled trials

**DOI:** 10.1371/journal.pone.0267534

**Published:** 2022-04-22

**Authors:** Basel Abdelazeem, Kirellos Said Abbas, Mostafa Atef Amin, Nahla Ahmed El-Shahat, Bilal Malik, Atefeh Kalantary, Mostafa Eltobgy

**Affiliations:** 1 McLaren Health Care, Flint, Michigan, United States of America; 2 Michigan State University, East Lansing, Michigan, United States of America; 3 Faculty of Medicine, Alexandria University, Alexandria, Egypt; 4 Faculty of Medicine, Cairo University, Cairo, Egypt; 5 Faculty of Medicine for Girls, Al-Azher University, Cairo, Egypt; 6 Adventist Health Simi Valley, Simi Valley, California, United States of America; 7 The Ohio State University, Columbus, Ohio, United States of America; The University of Mississippi Medical Center, UNITED STATES

## Abstract

**Background:**

Recruitment plays a vital role in conducting randomized control trials (RCTs). Challenges and failure of proper recruitment lead to early termination of trials. Monetary incentives have been suggested as a potential solution to these challenges. Therefore, we aimed to do a systematic review and analysis to evaluate the effect of incentives on the number of participants willing to consent to and participate in RCTs.

**Methods:**

Electronic databases were systematically searched from inception to September 23^rd^, 2021, using the following keywords: payments, incentive, response, participation, enrollment, randomized, randomization, and RCT. The Cochrane Risk of Bias tool was used to assess the quality of the included trials. Risk ratios (RRs) were calculated with their corresponding 95% confidence interval (CI). All analyses were done with the random-effects model. We used Revman software to perform the analysis.

**Results:**

Six RCTs with 6,253 Participants met the inclusion criteria. Our analysis showed significant improvement in response rate (RR: 1.27; 95% CI: 1.04, 1.55; P = 0.02) and consent rates (RR: 1.44; 95% CI: 1.11, 1.85; P = 0.006) when an incentive payment was offered to participants. Even a small amount of incentive showed significant improvement in both consent (RR: 1.33; 95% CI: 1.03, 1.73; P = 0.03) and response rates (RR: 1.26; 95% CI: 1.08, 1.47; P = 0.004).

**Conclusion:**

In conclusion, our meta-analysis demonstrated statistically significant increases in the rate of consent and responses from participants when offered even small monetary value incentives. These findings suggest that incentives may be used to reduce the rate of recruitment failure and subsequent study termination. However, further RCTs are needed to establish a critical threshold beyond which incentive amount does not alter response rates further and the types of RCTs in which financial incentives are likely to be effective.

## Introduction

Randomized controlled trials (RCTs) represent the most reliable evidence for clinical decision-making and evidence-based medicine [1]. Upon RCTs, high-quality meta-analyses can be conducted and inform evidence-based decision-making. Ethical and appropriate recruitment is vital to conducting high-quality RCTs upon which clinical guidelines and recommendations may be based. One of the challenges facing researchers conducting RCTs is participant recruitment for trials and the ethical considerations associated. The researchers may face obstacles in recruitment due to the outcome measured, the disease under investigation, or the efforts and time that the patients will spend to effectively participate in the trial [2, 3].

Despite the importance of recruitment for RCTs, a small number of studies have discussed how to address the practical and ethical challenges facing researchers attempting to recruit participants. One of the possible methods to facilitate recruitment is reward or incentives. Lack of reward and recognition was one of the identifiable factors which led to diminished participation rates in RCTs [4]. A Cochrane review recommended including monetary incentives as one of the effective strategies for recruiters [5]. Monetary incentives are ethically acceptable, as they may be viewed as a gesture of appreciation for the patient’s contributions, time, and effort.

A recent trial by Jennings et al. confirmed significant improvements in recruitment rates with incentives, but the total size of the increase was small [6]. Parkinson et al. recommended incentives as a method to improve recruitment and retention in trials in their recent checklist to develop proper recruitment and design in clinical trials [7]. On the other hand, Arundel et al. [8] reported that the financial incentive did not statistically significantly increase response rates. Therefore, our aim in this study was to assess the effect of financial incentives on the recruitment and participation of patients and to provide updated information regarding the impact of incentives on the number of participants willing to participate in an RCT. To the best of our knowledge, our article will be the first meta-analysis focused on monetary incentives and their effects on recruitment for RCTs.

## Methods

We followed Preferred Reporting for Systematic Review and Meta-Analysis (PRISMA) to conduct this systematic review and meta-analysis [9, 10] **S1 Checklist**.

### Data sources and search strategy

On September 23^rd^, 2021, five databases, PubMed, Scopus, Web of Science, Cochrane, and Embase, were systematically searched from inception using the following keywords; payments, incentive, response, participation, enrollment, randomized, randomization, and RCT. Detailed changes in the search terms according to each database are represented in the **S1 Table.** Results were imported into Covidence to start screening [11]. Related articles were researched in PubMed and Google Scholar to discover any potentially overlooked or missed articles.

### Study selection and eligibility criteria

Inclusion and exclusion criteria were described prior to the literature review. Initially, only the title and abstract were checked for relevance, and studies that did not meet the selection criteria were excluded. We reviewed the entire manuscript to ensure that the selected studies were suitable for our review.

We only included RCTs written in the English language that fulfilled the following criteria: (1) population: participants who were those approached to be enrolled or to give informed consent to participate in RCTs; (2) intervention: giving incentive in the form of monetary compensation; (3): comparison: no incentives were given; (4): assessing the effectiveness of incentives on participants to respond and/or give consents in RCTs. Three authors (MAA, NAE, and KSA) did the screening independently and were blinded to each other. A fourth author, BA, resolved any conflicts.

### Data extraction

Two authors (MAA and NAE) independently extracted the data, including the name of authors, year of publication, study design, country of the study, age of the participants, incentive value, study aim, and results. We extracted data about response rate and consent rate to analyze the pooled data. Additional data about response and consent rates were extracted according to the amount of incentive available. Any inconsistencies were settled by consensus among all investigators.

### Risk of bias assessment

Two authors, MAA and NAE, checked the quality of the included papers through The Cochrane Collaboration’s tool for assessing the risk of bias in randomized trials (ROB 1) tool [12]. The ROB tool assesses the quality of the trial for selection, attrition, detection, and reporting biases. Based on this assessment, each domain was assigned an overall risk of bias: Low, unclear, or high. The assessment was performed independently with discussion with KSA to resolve ambiguity or disagreements, as required.

### Outcomes of interest

The primary outcome was the response rate and/or consent rate to participate in RCTs. The secondary outcome was the subgroup analysis of the different levels of financial incentive and their influence on willingness to participate at each respective level.

### Statistical analysis

Meta-analysis of the pooled data was done through Revman v5.3 [13]. NAE and KSA analyzed the data, and it was reviewed by BA. Events and totals of responses and consents were plotted to calculate the Risk ratios (RRs). RRs were calculated with their corresponding 95% confidence interval (CI). All analyses were done with the random-effects model. Statistical tests were 2-sided, and P < 0.05 was considered significant. Subgroup analysis was performed on the value of incentives and their influence on the results. We considered $200 as a cut-off mark as the value was the median value between the financial value offered in the included RCTs. Therefore, studies were grouped into less than $200 or greater than $200. We analyzed each group separately to demonstrate the effect of each respective amount on the targeted outcomes. We did not perform publication bias analysis due to the small number of included RCTs [14].

## Results

### Search results and study selection

**Fig 1:**
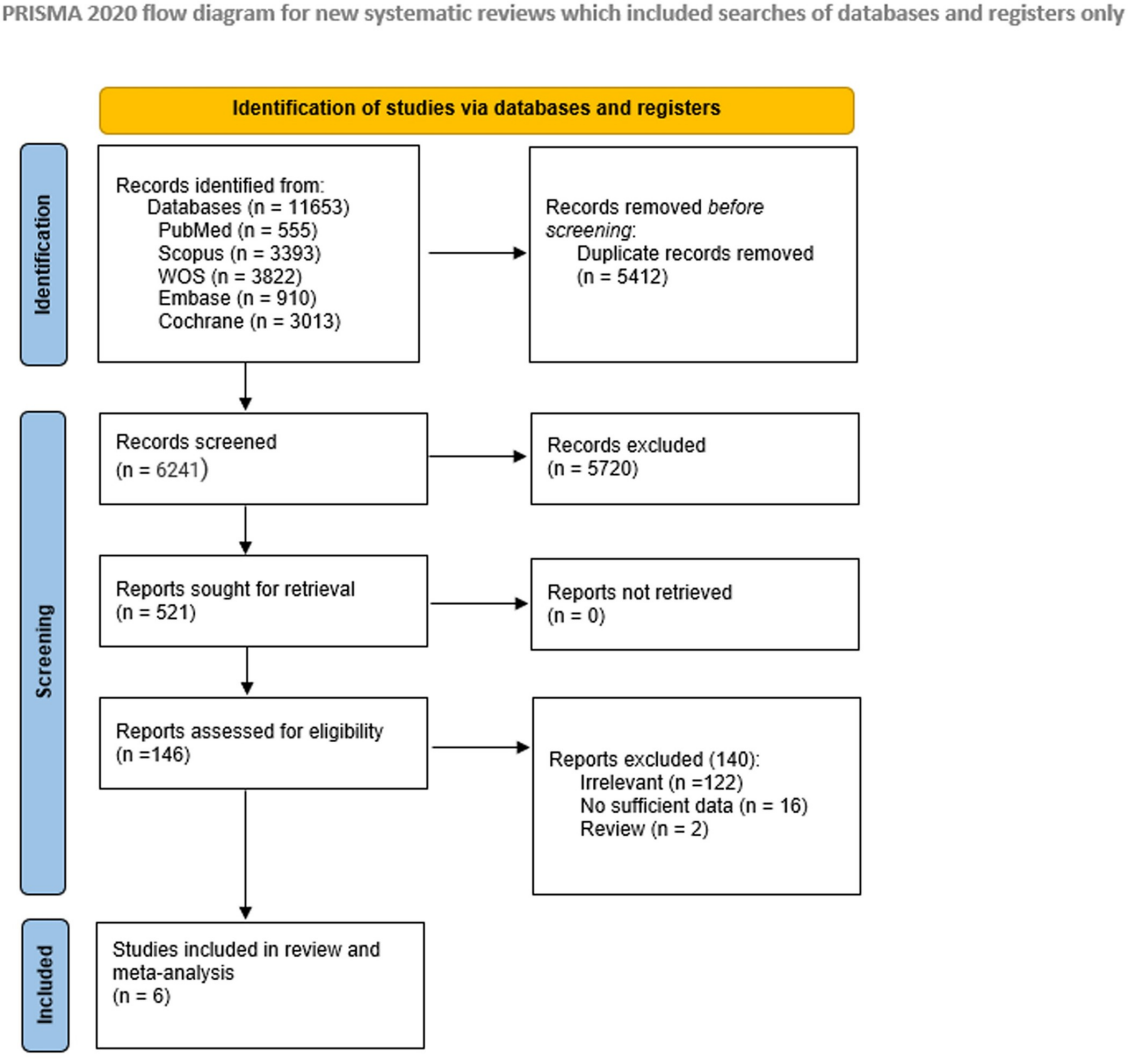
PRISMA 2020 flow diagram for updated systematic reviews, which included searches of databases, registers, and other sources.

Out of 11,653 studies imported from our databases’ search, 5,412 were automatically removed as duplicates by Covidence. A total of 6,241 were screened for title and abstract, and 521 for full-text screening. Six articles were ultimately included and analyzed in our study [6, 8, 15–19]. Halpern et al. reported two RCTs [18, 19] and published them in the same article [16]. **Fig 1** demonstrates the flow of our studies’ selection.

### Characteristics of included studies

All trials were from the USA, except one trial from Scotland [6]. Two were single-blinded [8, 15], and the rest of the RCTs were open-label [6, 16–19]. The amount of incentive received ranged from 2$ to 500$. Four RCTs were done for smoking cessation [8, 15, 17, 18] and one for ambulation among hospitalized patients [19]. A total of 6,253 participants were included and divided into the incentive group and control group. The participants’ age ranged from 15.5 years to 66.2 years. **Table 1** shows the characteristics of the included studies, as reported by the authors.

**Table 1:** Characteristics of the included studies.

Author, year	Country	Study design	Total population (n)	Gender, male (n)	Mean age (years)	Incentive value	Condition of incentive	Response meaning	Study environment	Results
Martinson et al. 2000 [17]	USA	Open label RCT*	3,038	1,519	15.5	Prepaid 2$ with survey And 15$	Completion of the survey and return	The specific response rate to the survey, Subject consent to further contact	Smoking cessation.	Incentives increased response rates (55% response without incentive vs. A 69% response with incentive), with a response of 74% in the $15 cash group 69% in the token group.
Arundel et al. 2019 [8]	USA	Single-blinded RCT	423	NR	NR	20£	NR	Completing a CO breath measurement	Smoking Cessation Intervention for severe Mental Ill Health Trial (SCIMITAR+).	Contingent financial incentives did not statistically significantly increase rates of face-to-face follow-up completion.
Jennings et al. 2015 [6]	Scotland	Open-label RCT	1015	589	66.2	100£	After coming screening visit and signing the consent	Response rates to the first invitation letter, a screening visit	One of five of SCOT, FAST, and PATHWAY 1, 2, and 3 trials.	A £100 incentive payment offer led to small but significant improvements in patient responses to a clinical trial invitation letter.
Free et al. 2010 [15]	USA	Single-blinded RCT	491	NR	35.9	Incentive value	Condition of incentive	Response meaning	Smoking cessation support.	A 5.3% (13/246) of participants who were sent the letter with £5 gave their consent to join the trial, compared with 0.4% (1/245) of the control group.
Halpern et al. 2021 (NCT02378714) [16, 18]	USA	Open-label RCT*	654	251	50.6	Prepaid 2$ with survey And 15$	Completion of the survey and return	The specific response rate to the survey, Subject consent to further contact	Smoking cessation.	Consent rates were 21.8%, 35.9%, and 47.1% in the $0, $200, and $500 arms, respectively
Halpern et al. 2021 (NCT03321279) [16, 19]	USA	Open-label RCT*	632	278	46.7	100$ and 300$			Ambulation among hospitalized patients.	Consent rates were 45.4%, 48.1%, and 43.0% in the $0, $100, and $300 arms

N: number; NR; Not reported; $: dollar; £: pound; RCT: randomized control trial; USA: United States of America; SCOT: Standard care versus Celecoxib Outcome Trial; FAST: Febuxostat versus Allopurinol Streamlined Trial; PATHWAY 1,2,3: three British Heart Foundation-funded Prevention and Treatment of Hypertension with Algorithm Guided Therapy

*The RCTs has three groups of comparison

### Risk of bias of the included studies

**Fig 2:**
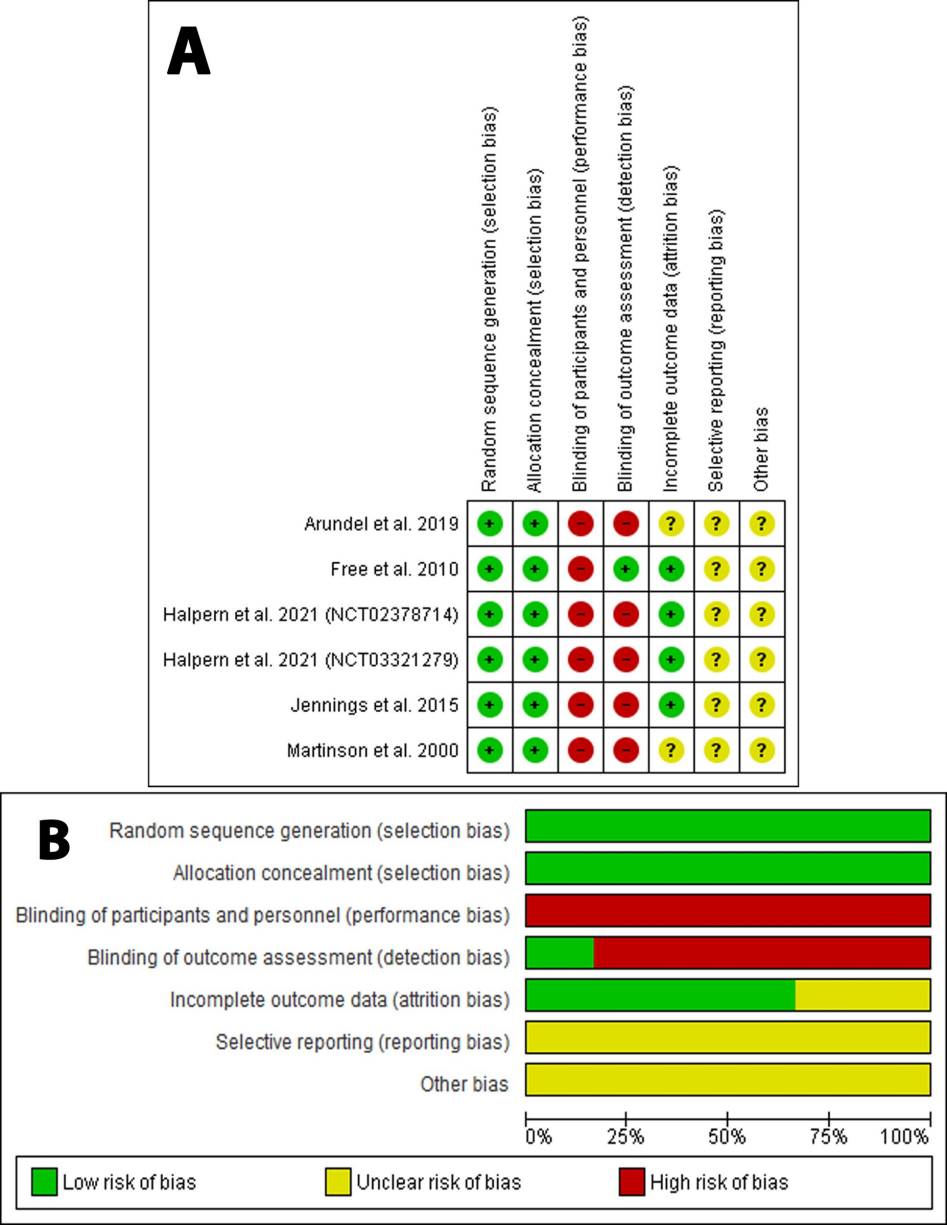
Risk of bias assessment. **A**: Risk of bias summary: review authors’ judgments about each risk of bias item for each included study. **B:** Risk of bias graph: review authors’ judgments about each risk of bias item presented as percentages across all included studies. The items are scored (+) low risk; (-) high risk; (?) unclear risk of bias.

Overall assessment of the included studies did not consider poor-quality studies. However, all studies demonstrated a high risk of bias in performance bias [6, 8, 15–19]. All of the studies [6, 8, 16–19] showed a high risk of bias in detection bias categories except Free et al. [15]. All the studies demonstrated a low risk of bias in the randomization process and selection bias. All studies showed uncertain reporting bias and any other biases. Details of the assessment are illustrated in **Fig 2.**

### Outcomes of interest

**Fig 3:**
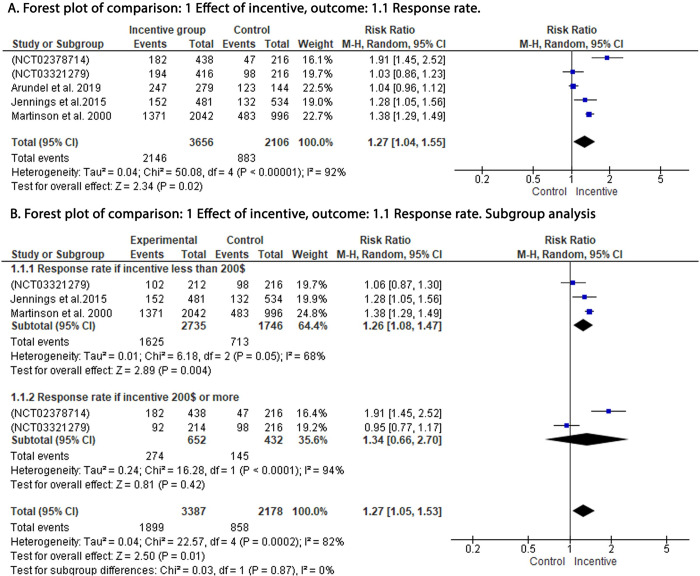
Forest plot of the effect of incentive on the response rate. A: Forest plot of the effect of incentive on the response rate. B: Subgroup analysis for the effect of incentive on the response rate. df: degrees of freedom; I^2^: I-squared; M-H: Mantel-Haenszel variance; CI: confidence interval.

**Fig 4:**
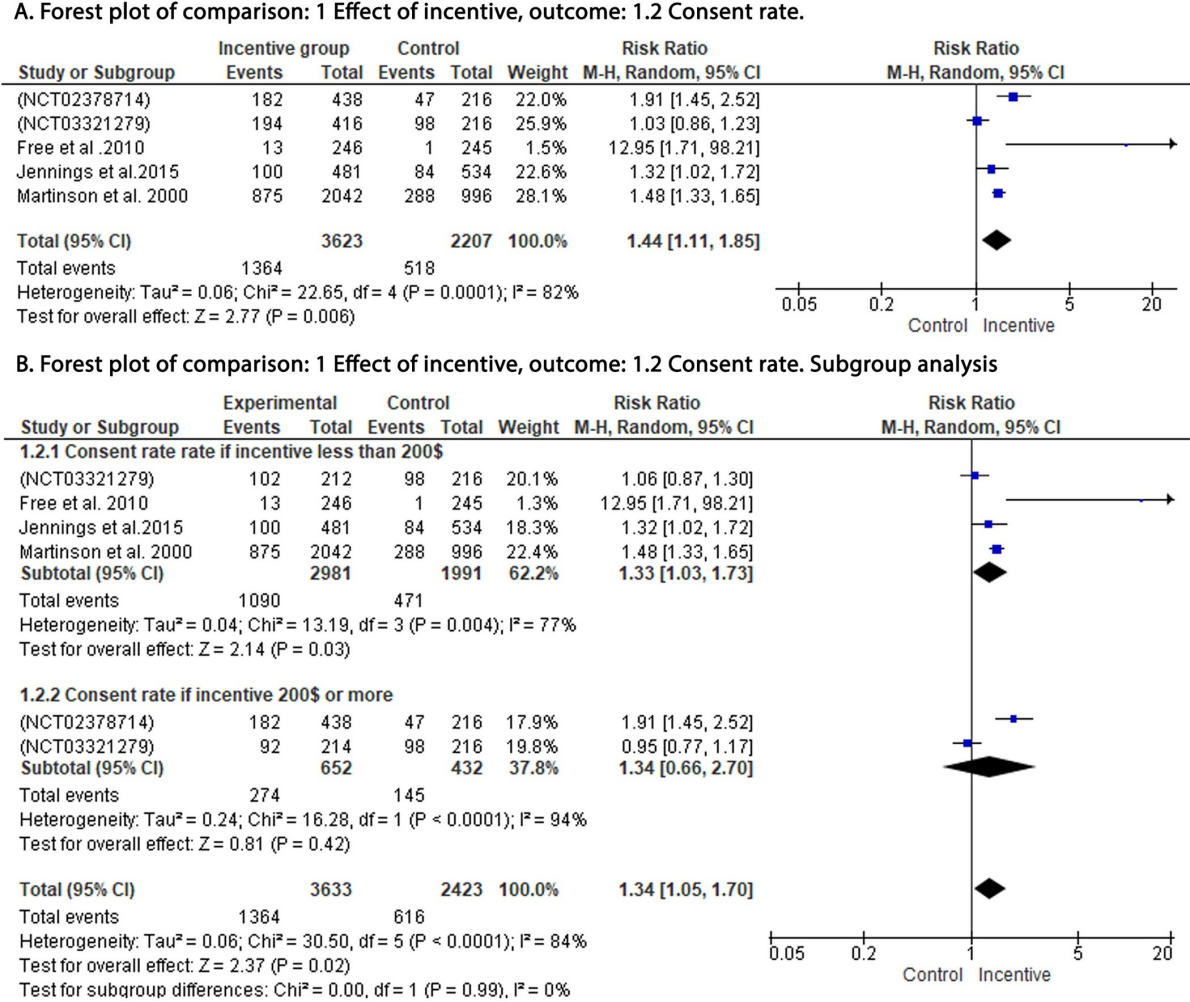
Forest plot of the effect of incentive on the consent rate. A: Forest plot of the effect of incentive on the consent rate. B: A: Subgroup analysis for the effect of incentive on the consent rate. df: degrees of freedom; I^2^: I-squared; M-H: Mantel-Haenszel variance; CI: confidence interval.

Our analysis showed significant improvement in response rates (RR: 1.27; 95% CI: 1.04, 1.55; P = 0.02) **Fig 3A** and consent rates (RR: 1.44; 95% CI: 1.11, 1.85; P = 0.006) with the use of incentives when compared to the control group **Fig 4A.**

Subgroup analysis (**Figs 3B and 4B)** showed that the incentives of less than $200 were still associated with significant improvement in response rates (RR: 1.26; 95% CI: 1.08, 1.47; P = 0.004) and consent rates (RR: 1.33; 95% CI: 1.03, 1.73; P = 0.03). Analysis of studies with incentives of $200 or more did not demonstrate significant improvement in both response rates (RR: 1.34; 95% CI: 0.66, 2.70; P = 0.42) and consent rates (RR: 1.34; 95% CI: 0.66, 2.70; P = 0.42).

## Discussion

The recruitment process for RCTs has posed a great challenge and has been historically a cause of early study termination. Our study shows a significant increase in the number of consents and response rate with monetary incentives. Even with a small amount of financial incentive, there were still significant improvements in the outcomes measured. However, we found insignificant results with more than $200, which may be attributed to the small number of included studies and participants or due to the perceived risk or the nature of the intervention itself. Therefore, the current data is not sufficient to draw conclusions regarding the ideal cut-off for financial incentives relative to maximal improvements in response and retention rates. To the best of our knowledge, this review provides the most updated data on the effect of monetary incentives on RCT participation available in the current literature.

Further studies are required to clarify the ideal recruitment and incentivization strategies for particular study types, as the literature does suggest that financial incentives may not change the willingness of participants to join the study in certain instances [16]. Other factors influencing trial participant retention must also be considered in the overall trial design. Patients find the trial process demanding or strenuous, especially when requiring significant amounts of time and implicating other financial costs [15]. Potential loss of participation may also occur when there is unfavorable randomization, for example, if the patient wanted a certain allocation but received another, difficult protocols or boring protocols, the need for further testing or interventions per trial requirements, and lack of understanding of the reason the trial is being conducted [15]. Other factors include loss of patients and recruiter motivation [20]. Many of the reasons leading to a potential loss of participation can be mitigated by providing financial incentives, which would increase the willingness of patients to tolerate tedious or boring protocols.

Throughout the literature, there were concerns regarding patients who were socially deprived, elderly, and those with a high number of comorbidities may be poorly represented during the recruitment process [6]. This would limit the applicability of the study to the general population due to sampling biases [6, 21]. With financial incentives being in place, several studies [21–24] have suggested that the opposite concern may also come into play, whereby lower socioeconomic status (SES) participants would be more inclined to participate than those from a higher SES. Lower SES individuals may rely on the incentives from trials and similar opportunities as a source of direct income, leading to more influence on their decision-making regarding participation [21]. Ultimately, the provision of financial incentives for participation may alter the participants’ responses or shift the study’s demographic, and this impact should be monitored and accounted for in future trials.

The implications of financial incentives influencing decisions, particularly in lower SES classes, carry serious ethical considerations. Generally, education levels tend to be lower amongst these populations, and the interventions proposed by a given study may be beyond the understanding of certain participants. Another possible study area would include the willingness to participate in patients before and after in-depth education regarding interventions, risks, benefits, and potential implications on quality of life in lower SES populations. These ethical considerations are generally addressed by institutional review boards (IRB) processes on a systemic level, but further consideration is warranted due to areas of possible ambiguity. For example, if an IRB committee approved a study involving a high-risk intervention, financial incentives, and a complete written explanation of the intervention that involved recruitment from populations with lower SES and education levels. The possibility that participants may not fully comprehend the written language or concepts may easily be overlooked.

Further, Resnik et al. suggested that the investigators should stratify payments by income level or SES, considering higher payments to those with lower SES [21]. This may become a tricky situation, whereby it could be argued that it is unethical to pay two individuals differently for the same work based on their SES. Conversely, not providing any financial incentive or compensation for study participation may also bias the results as those with fewer means may be less able to afford to participate. These concepts demonstrate the importance of improving recruitment practices to involve a greater breadth of patients to improve the applicability of any given study. This is especially important in trials that have the potential to influence major medical decision-making and guidelines for the same. Interestingly, Halpern et al. 2021 described differences amongst the successes of financial incentives in improving recruitment rates amongst different types of RCTs. For example, these incentives improved willingness to participate in a trial involving smoking cessation but not an RCT involving ambulation in hospitalized patients. However, a larger incentive was offered in the ambulation trial compared to the smoking cessation ($300 and $200, respectively) [16].

The following areas of interventions should be carefully evaluated to improve the recruitment process before conducting the RCTs: trial design, informed consent, approach to participants, delivery of trial information, and training for recruiters. Using an open-labeled design, in which patients are not blinded to interventions, results in a higher recruitment rate [25]. Limitations of improving recruitment based on open-label studies include the increased risk of biases associated with the loss of blinding. Treweek et al. [5] found that the opt-out option in the consent and the possibility to leave the trial upon need showed better results than the opt-in option in the consent. Delivering information about the trial through more interactive computer-based presentations and audio- presentations also showed a slight improvement in recruitment. Additional training for recruiters did not improve recruitment overall. Caldwell et al. reported a significant increase in the number of consents with increasing awareness of the impact of the trial on participants’ health and medical practice in general [26].

Financial incentives can increase the recruitment and retention of underrepresented groups like minorities and the socioeconomically disadvantaged. Researchers should be aware of the individual barriers that can face these groups when attempting to participate in research and propose some solutions. Another significant barrier to recruitment included the mistrust of the government and research as an overall practice. Patient education with regards to the process of formulating a research project and bringing it to fruition (IRB, reviews, patient consent practices, etc.) may be a possible solution for addressing some of these concerns. Transportation is another potential obstacle to participation for those without stable means. A possible solution is to provide basic transport to and from participants’ locality. Finally, economic and time restraints are significant barriers. Flexibility regarding participation timing and monetary incentives can reduce the impact in these scenarios [27].

Our study has a few limitations. First, only a few RCTs were included in our study, and more RCTs are needed to increase the validity and accuracy of our results. Currently, there is an ongoing study investigating the recruitment patterns of participants and predicting factors influencing patients’ willingness to participate [28]. Second, the heterogeneity between the included RCTs was high due to variation in the intervention, which may impact the generalizability of our results. Third, the included RCTs did not evaluate therapeutic misconceptions and perceived coercion in patients who decided not to participate in the RCTs. Fourth, the included RCTs were of low-risk intervention; therefore, trials with high-risk intervention, such as investigating the treatment of severe illness or invasive procedure, should be conducted to evaluate the internal validity of our results.

## Conclusion

In conclusion, our meta-analysis demonstrated statistically significant increases in the rate of consent and responses from participants when offered even small monetary value incentives. These findings suggest that incentives may be used to address the issues of recruitment failure and subsequent study termination. However, further RCTs are needed to establish a critical threshold beyond which incentive amount does not alter response rates further and the types of RCTs in which financial incentives are likely to be effective.

## Supporting information

S1 Checklist PRISMA 2020 checklist. Preferred Reporting Items for Systematic Review and Meta-Analysis.
(DOCX)

S1 Table Search terms and results in different databases. (DOCX)
