# Supplementary material for: The effectiveness of incentives for research participation: A systematic review and meta-analysis of randomized controlled trials
Source: PLoS One. 2022 Apr 22;17(4):e0267534. doi: 10.1371/journal.pone.0267534 (PMC9032371; doi:10.1371/journal.pone.0267534)
Supplement: S1 Table — (DOCX) [file pone.0267534.s002.docx]

| Database | Search term | Results |
| --- | --- | --- |
| PubMed | (randomized[Title/Abstract] OR randomised[Title/Abstract] OR random[Title/Abstract] OR randomly[Title/Abstract] OR randomization[Title/Abstract] OR randomisation[Title/Abstract] OR RCT[Title/Abstract] OR RCTs[Title/Abstract]) AND (Payments[Title/Abstract] OR incentive[Title/Abstract]) AND (response[Title/Abstract] OR particip*[Title/Abstract] OR enroll*[Title/Abstract]) | 555 |
| Web of science | (Payments OR incentive) AND (response OR particip* OR enroll*) AND (randomized OR randomised OR random OR randomly OR randomization OR randomisation OR RCT OR RCTs) | 3,822 |
| Scopus | TITLE-ABS ( payments OR incentive ) AND TITLE-ABS ( response OR particip* OR enroll* ) AND TITLE-ABS ( randomized OR randomised OR random OR randomly OR randomization OR randomisation OR rct OR rcts ) | 3,353 |
| Embase | #4: #1 AND #2 AND #3 Results: 910 #1: payments OR incentive Results: 33,759  #2: response OR participation OR enrollment Results: 4,211,117  #3: randomized OR randomised OR random OR randomly OR randomization OR randomisation OR rct OR rcts Results: 1,963,802 | 910 |
| Cochrane | randomized OR randomised OR random OR randomly OR randomization OR randomisation OR rct OR rcts in All Text AND response OR participation OR enrollment in All Text AND payments OR incentive in All Text | 3,013 |

Table S1. Search terms and results in different databases
